# Current levels of coverage of iron and folic acid fortification are insufficient to meet the recommended intake for women of reproductive age in low- and middle-income countries

**DOI:** 10.7189/jogh.11.18002

**Published:** 2021-10-02

**Authors:** Hanzhi Tong, Neff Walker

**Affiliations:** Department of International Health, Bloomberg School of Public Health, Johns Hopkins University, Baltimore, Maryland, USA

## Abstract

**Background:**

Food fortification can be an effective intervention to improve maternal and child health. Folic acid fortification can reduce neural tube defects due to folate deficiency. Iron fortification is effective to reduce maternal anemia due to iron deficiency. The paper describes the methods for estimating current coverage levels for iron fortification and folic acid fortification and estimates current impact of fortification in low- and middle-income countries (LMICs) using the Lives Saved Tool (LiST).

**Methods:**

The database was obtained from Global Fortification Data Exchange. We used the following indicators from the database: food intake, fortification standard, percent of food produced in industrial mills, and percent of industrially milled food that is fortified. Together with the recommended dietary allowances for women of reproductive age (WRA), we calculated percentage of WRA getting recommended intake through fortification and used the percentage as an estimate for fortification coverage. We then used LiST to estimate the health impact of fortification on maternal and child health.

**Results:**

Folic acid was fortified in 72 countries, with a median coverage of 43%. Iron was fortified in 87 countries, with a median coverage of 23%. Forty-six LMICs fortified either folic acid, iron, or both. And the weighted coverage of folic acid fortification and iron fortification were 34% and 19%, respectively. A greater percentage of WRA got appropriate levels of folic acid and iron via fortification in higher income countries. Based on LiST projection, it is estimated that in 2021, over 4 million anemia cases among WRA will be averted due to consumption of iron fortified food. About 1900 stillbirths and 3000 neonatal deaths due to neural tube defects will be averted due to consumption of folic acid fortified food.

**Conclusions:**

We estimated the coverage of folic acid fortification and iron fortification in LMICs and included them in the most recent version of LiST. Trends in coverage will be included in LiST as data become available. Our analysis shows that while most LMICs have fortification programs, currently the effects of these programs are limited either through low levels of fortification in industrialized food, low consumption of fortified food or both.

Micronutrient deficiency is significantly associated with the global burden of various diseases and the prevalence is especially high in low- and middle-income countries. Pregnant and lactation women and children are the most vulnerable [1,2]. Pregnant women with severe anemia have double the risk of mortality than non-anemic women [3]. The major cause of anemia – iron deficiency remained one of the most common micronutrient deficiencies [4]. In 2015, an estimated 260 100 birth outcomes were affected by neural tube defects (NTDs) and about 75% of the NTD-affected births resulted in under-five mortality [5]. Many of these birth defects could be prevented with folic acid (vitamin B9) [6]. Iron and folic acid supplementation are recommended to all pregnant women, aiming to reduce maternal anemia and impaired birth outcomes.

One possible approach to reducing nutritional deficiencies is through food fortification. Studies have shown the effectiveness of food fortification to reduce micronutrient malnutrition [7]. The global control of iodine deficiency – halved over the past decade, due to iodized salt is a good example. The intervention is a low cost (US$ 0.05 per person per year) and universally acceptable because salt is widely available and consumed globally [8]. Fortification can become an important element within the food system, as well as public health. The key to effectiveness of fortification depends on both getting processed foods fortified and the extent to which these foods are part of the diet of the population.

In Lives Saved Tool (LiST), we aimed to capture a comprehensive list of interventions that can reduce mortality among women, neonates and children [9]. The paper describes the inputs and methods for estimating coverage levels for iron fortification and folic acid fortification in low- and middle-income countries (LMICs) two interventions that are used in LiST. We then use the LiST model to estimate yearly and five-year health benefits to women and children due to current levels of consumption of iron and folic acid fortified foods.

## METHODS

We developed method to estimate country-specific coverage of iron fortification and folic acid fortification using database from Global Fortification Data Exchange (GFDx). GFDx uses a combination of primary and secondary data to populate the database [10]. The primary data are collected via surveys sent out by GFDx stewards: Food Fortification Initiative, Global Alliance for Improved Nutrition, and Iodine Global Network, to countries included in the GFDx. The secondary data are retrieved via online desk review or partner databases, such as Food supply database from Food and Agriculture Organization (FAO) [11]. The database includes fortification information of five food and condiment vehicles: wheat flour, maize flour, rice, oil, and salt.

The database also includes indicators related to legislation, fortification standards, and industry organization. The full database is retrieved from https://fortificationdata.org/. To estimate the coverage of iron and folic acid fortification, we use the following indicators: fortification standard (µg/g), food intake (g/c/d), percent of food produced in industrial mills, and percent of industrially milled food that is fortified. We will calculate coverage for countries with complete data on all four indicators. We did not have mandatory fortification as an inclusion criterion in our calculation of coverage estimate, because we believe that even voluntary fortification can lead to certain degree of fortification coverage.

The food fortification standard, if any, is defined as nutrient level in µg per gram of the food vehicle. For folate, folic acid is the only form of compound fortified. For iron fortification, various forms of iron compounds are used, ranging from NAFeEDTA, elemental iron, to ferrous sulfate. If one food vehicle can be fortified by different forms of iron, only one fortification standard is included in calculation, assuming similar impact of various forms to avoid overestimation. Fortification standard in different countries were implemented in different years, between 2010 to 2018. We use the most recent fortification standard. Seventy-seven and Ninety-three countries have fortification standards for folic acid fortification and iron fortification, respectively.

Food intake data are defined as average daily intake in gram per person. FAO provided the trend of food intake overtime. We used the data from 2017, which is the most recent data available. The food intake data were missing for six countries, including two LMICs, that have iron or folic acid fortification standard.

The percent of food produced in industrial mills and percent of industrially milled food that is fortified are used as compliance data. Most of the compliance data are from 2017 and 2018.

**Figure 1:**
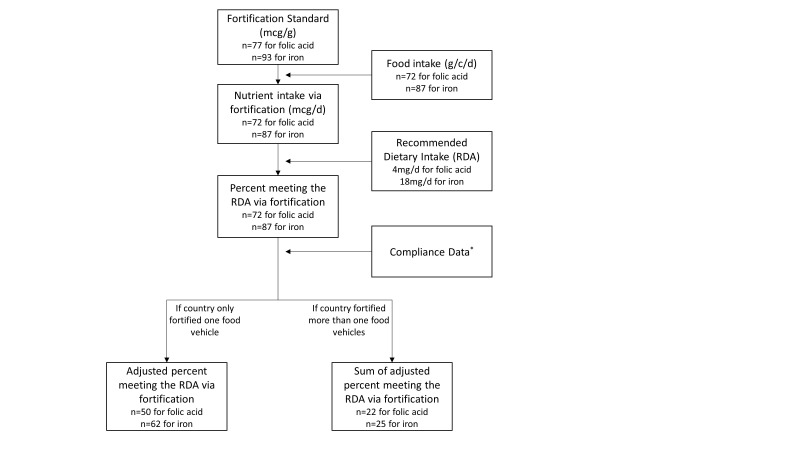
Steps to estimate the coverage level for iron and folic acid fortification. RDA – recommended dietary intake of iron and folic acid for women of reproductive age, n – number of countries. *Compliance data are the percent of food vehicle that are industrially processed and the percent of industrially processed that are fortified.

**Figure 1** demonstrates the steps of estimating coverage of iron fortification and folic acid fortification in 2018. First, assuming all consumed foods are fortified: nutrient intake via fortification (µg/d) = food intake (g/c/d) × fortification standard (µg/g).

Second, we compared nutrient intake via fortification to the recommended dietary allowances (RDA) for women of reproductive age: 4mg/d folic acid and 18mg/d iron. Percent meeting the RDA = nutrient intake via fortification/recommended nutrient intake. If the fortified nutrient intake exceeds the recommended intake, the percentage is topped at 100%, interpreted as the nutrient intake via fortification fully met the recommended intake.

Third, we adjusted for compliance data: adjusted percent meeting the RDA = percent meeting the RDA × percent industrially processed × percent industrially processed food that is fortified. The adjusted percent meeting the RDA are used as the coverage level for fortification.

The fourth step is optional for countries that fortified more than one food vehicle, the sum of adjusted percent meeting the RDA for all fortified food vehicles are used as the coverage level for fortification.

Indicators are extracted into Excel (Microsoft Inc, Seattle WA, USA) and all the calculations are performed using Excel (Microsoft Inc, Seattle, WA, USA). Due to the lack of data availability on nutrient intake through diet, our definition of fortification coverage is defined as percentage of WRA getting recommended intake through fortification. Our definition is the minimum percentage of coverage because we did not count for iron and folic acid intake from diet other than staple foods. The countries with no data were assumed to have no fortification therefore the coverage level is zero percent.

We also retrieve the 2018 population data to estimate weighted coverage of fortification among LMICs providing fortification and percent of the population in LMICs covered by fortification [12]. LMICs are low- and lower-middle income countries defined by World Bank classification [13]. To estimate the health impact of iron and folic acid fortification over a one-year period and a five-year period in LMICs, we use LiST, a modeling tool which allows users to estimate the impact of coverage change of interventions on maternal, neonatal and child health. The methodology is explained in a 2013 paper by Walker et al (9). LiST has been widely used for strategic planning, evaluation, and advocacy. Since nutrition plays an important role in maternal and child health outcomes, LiST has expanded to include a variety of nutrition-related interventions that target stunting, maternal anemia, birth outcomes, breastfeeding and wasting [14].

In LiST iron and folic acid fortification has two main effects. For iron fortification the main effect is in reducing anemia in women. This is both for women of reproductive age and for pregnant women. In LiST, we assume that iron fortification can reduce anemia due to iron-deficiency by 34% among all women of reproductive age [7]. Note that the rates of anemia and percent of anemia due to iron deficiency are country specific [4].

Folic acid fortification has its primary effect on the development of the fetus of pregnant women. Folic acid has a protective effect in reducing the risk of neural tube defects, which in turn reduces the risk of still births as well as early neonatal deaths. Specifically, folic acid fortification can reduce 41% stillbirth due to NTDs [15] and 41% neonatal mortality due to NTDs [7].

We use LiST to create projections from 2017 to 2022 for each LMIC where there is either fortification with iron, folic acid or both started in 2018. Holding all the other country data constant, we generate the estimates of the effects of fortification: number of anemia cases, stillbirth and neonatal mortality averted.

## RESULTS

We calculated folic acid fortification coverages for 72 countries with completed data, including 43 LMICs (**Table 1**). Wheat flour was most commonly fortified food, followed by maize flour and rice. The coverage ranged from 0% to 100%, with a median of 43%. The median coverages in Africa region and South-East Asia region were 28% and 3%, respectively. The weighted folic acid fortification coverage by population size in the 43 LMICs was 34%.

**Table 1:** Median iron and folic acid fortification coverage by income status and by WHO region

	Folic acid fortification			Iron fortification		
	**Median coverage (%)**	**IQR**	**Number of countries**	**Median coverage (%)**	**IQR**	**Number of countries**
Income status:						
High	85	16	10	42	24	15
Upper Middle	73	37	19	37	26	26
Lower Middle	36	42	26	18	29	29
Low	21	14	17	8	9	17
WHO Region:						
Africa	28	48	24	11	13	26
Americas	60	49	21	34	26	33
Eastern Mediterranean	90	35	11	45	38	12
European	59	56	4	54	53	4
South-East Asia	3	32	5	2	18	5
Western Pacific	7	82	7	0.2	18	7

WHO – World Health Organization, IQR – interquartile range

We calculated iron fortification coverage for 87 countries with completed data, including 46 LMICs (**Table 1**). Wheat flour was most commonly fortified food, followed by maize flour, rice, and salt. The coverage ranged from 0% to 100%, with a median of 23%. The median coverages in Africa region and South-East Asia region were 11% and 2%, respectively. The weighted iron fortification coverage by population size in the 46 LMICs was 19%.

The average compliance (% industrially processed * % industrially processed food that is fortified) to the fortification of wheat flour were 93%, 73%, 66%, and 62% in high-, upper middle-, lower middle-, and low-income countries, respectively. Nineteen countries, including 10 LMICs, had zero compliance for one or two of the fortified food vehicles (wheat flour, maize flour, rice, and salt).

A greater percentage of women got appropriate levels of folic acid and iron via fortification in higher income countries. Median folic acid fortification coverages were 85%, 73%, 36%, and 21% in high-, upper middle-, lower middle-, and low-income countries, respectively. Median iron fortification coverages were 42%, 37%, 18%, and 8% in high-, upper middle-, lower middle-, and low-income countries, respectively. The coverage levels also varied in six WHO regions (**Table 1**). Countries in Americas, Eastern Mediterranean, and European regions had higher coverages for both nutrients. The coverages were relatively low in Western Pacific countries, with a median coverage of 7% for folic acid and 0.2% for iron. For country-specific fortification coverages, please go to LiST online (https://list.spectrumweb.org/) or download the Spectrum (https://www.livessavedtool.org/listspectrum).

The 46 LMICs that fortified either folic acid, iron, or both, covered approximately 88% of the total population in LMICs. In 2021, 4 155 290 cases of anemia among WRA will be averted due to consumption of iron fortified food. And 1943 stillbirths and 2987 neonatal deaths will be avoided due to consumption of folic acid fortified food. The potential health benefits of fortification for women and children by region in 2021 were presented in **Table 2**. When we looked at the five-year period right after the fortification started in 2018, 20 410 883 cases of anemia among WRA can be averted due to iron fortification. And 9578 stillbirths and 14 780 neonatal deaths can be avoided due to folic acid fortification. The five-year impacts by region were presented in **Table 3**.

**Table 2:** Health benefits of fortification for women and children by WHO regions in 2021

WHO region	Anemia cases averted among WRA	Stillbirths averted	Neonatal deaths averted
Africa	1 200 161	1,293	1,651
Americas	86 668	9	52
Eastern Mediterranean	1 252 620	429	645
European	432 425	23	152
South-East Asia	893 072	188	485
Western Pacific	290 344	1	2
Total	4 155 290	1943	2987

WHO – World Health Organization, WRA – women of reproductive age

**Table 3:** Health Benefits of fortification for women and children by WHO regions over a five-year period (2018-2022)

WHO region	Anemia cases averted among WRA	Stillbirths averted	Neonatal deaths averted
Africa	5 843 983	6348	8118
Americas	427 560	45	258
Eastern Mediterranean	6 129 866	2117	3184
European	2 139 493	117	767
South-East Asia	4 441 114	946	2443
Western Pacific	1 428 867	5	10
Total	20 410 883	9578	14 780

WHO – World Health Organization, WRA – women of reproductive age

## DISCUSSION

Using the GFDx database, we define the coverage of fortification as the percent of WRA getting recommended intake via fortification. Currently, 46 LMICs which represents about 88% of the population in all LMICs had fortification standard for either iron, folic acid, or both. However, both the weighted folic acid fortification and iron fortification coverage by population size were suboptimal, 34% for folic acid and 19% for iron. Based on the LiST projection, it is estimated that over 4 million anemia cases can be averted in 2021 due to current level of consumption of iron fortified food. And with the current level of consumption of folic acid fortified food, about 1900 stillbirths and 3000 neonatal deaths due to NTDs can be avoided in 2021.

There was large variation in coverage of iron and folic acid fortification in LMICs. Wheat flour was the most commonly fortified grains. The food intake depends on the consumption pattern. Rice is more commonly consumed in Asian countries, while maize flour is more common in African countries. The fortification standards also varied across different countries: ranging from 0.1 μg/g in India (wheat flour) to 5.11 μg /g in Vietnam (wheat flour) and 10 μg /g in Ethiopia (salt) to 975 μg /g in India (salt) for folic acid and iron, respectively.

The major cause for variation of coverage was due to the difference in compliance. A country can have sufficient fortification standard for proper food vehicle(s) to reach an ideal nutrient intake via fortification that fully met the RDA but if the compliance is low, meaning either small portion of the food vehicle are industrially processed or a lot of the industrial mills did not follow the fortification standard, the actual nutrient intake is low and subsequently, the coverage level is low. For countries currently with zero compliance, coverage of fortified foods could be greatly increased if levels of fortification in industrialized food were increased. It was estimated that among 18 LMICs with greater burden of anemia and neural tube defects, if all the industrial milled food are fortified, 72.1 million cases of anemia among WRA and 46 378 child deaths associated with NTDs can be averted annually [16].

There are some limitations of the study. Since the coverages are estimated based on secondary analysis of the open access database, it is hard for us to assess the quality of the data inputs. We mainly relied on information provided by GFDx and its steward to ensure the validity of the data. In addition, we do not have detailed information on how the countries define various indicators, especially on the percent of industrially processed food that are fortified, there might be variation between countries to check this compliance data. Due to lack of more background information, we assume the indicators are harmonized across countries.

One strength of our analysis is that we considered the target population of iron and folic acid fortification program when estimating the coverage. Since both nutrients are linked to outcomes related to WRA and WRA have a higher daily need, we did not use the WHO recommendations on wheat and maize flour fortification for the general population [17]. Instead, we compare the potential nutrient intake via fortification with recommended intake for WRA.

In addition, we recognized that in LMICs, a portion of grains are produced at farms and consumed without any processing. We also recognized that even within the centralized production, it is impossible to reach 100% fortification. Therefore, we include compliance data in our estimate for coverage to reflect situation in the real world. Since WRA had a higher daily need for both iron and folic acid and many LMICs has low compliance, our estimated impact of coverage might be lower than previously estimated coverage when using international standard and without adjustment for compliance, where it was estimated that 65 380 neural tube defect cases were prevented in a year [18].

## CONCLUSION

Defining coverage as percent of WRA meeting the RDA via fortification, we used GFDx database to estimate the coverage of folic acid fortification and iron fortification in low- and middle-income countries in 2018 and included them in the most recent version of LiST. We will include the future trend when data become available.

Our analysis shows that while most low- and middle-income countries do have fortification programs, currently the effects of these programs are limited either through low levels of fortification in industrialized food, low consumption of fortified food or both. Since both iron and folic acid play an important role in maternal, newborn and child health, including their fortification coverage in LiST helps to estimate the long-term impact of fortification on a public health level.
